# The association between blood nickel level and handgrip strength in patients undergoing maintenance hemodialysis

**DOI:** 10.1007/s11255-023-03836-2

**Published:** 2023-10-18

**Authors:** Ruiying Tang, Jiexin Chen, Huijuan Ma, Jihong Deng, Yanxia Zhang, Qingdong Xu

**Affiliations:** Department of Nephrology, Jiangmen Central Hospital, Jiangmen, China

**Keywords:** Blood nickel, Handgrip strength, Malnutrition, Hemodialysis

## Abstract

**Background:**

Progressive loss of peripheral muscle strength is highly pronounced in patients receiving maintenance hemodialysis (MHD), of which the pathological mechanism tends to be multifactorial. Plasma nickel was reportedly correlated with muscular strength in non-dialysis patients. However, scarce is known regarding the association between blood nickel level and handgrip strength among the patients undergoing MHD.

**Methods:**

This cross-sectional study included patients undergoing MHD at our center in October 2021. Blood samples were collected before the hemodialysis sessions. Nickel level was measured using inductively coupled plasma mass spectrometry. Eligible patients were stratified into three groups by the blood nickel level: tertile 1 (≥ 5.2 ug/L); tertile 2 (< 5.2 ug/L and ≥ 4.5 ug/L); and tertile 3 (< 4.5 ug/L). Handgrip strength measurement was used to evaluate the muscle status. Spearman’s analyses and multivariable linear regression analyses were performed to study the relationship between blood nickel level and handgrip strength.

**Results:**

A total of 236 patients were enrolled, with an average age of 55.51 ± 14.27 years and a median dialysis vintage of 83 (IQR: 48–125) months. Patients in group with a higher blood nickel level (tertile 1) tended to be female, had longer dialysis vintage and higher Kt/V, but lower BMI, serum creatinine, hemoglobin, and handgrip strength level (all *p* < 0.05). After adjustment for confounding factors in multivariable models, for every 1ug/L increase in nickel level, the patient’s handgrip strength decreases by 2.81 kg (*β*: − 2.810, 95% confidence interval: − 5.036 to − 0.584, *p* = 0.014). Restricted cubic spline confirmed the relationship was nearly linear.

**Conclusions:**

Our study highlighted that blood nickel level was related to handgrip strength in patients undergoing MHD. Prospective studies with larger sample sizes are still needed to confirm the result.

## Introduction

As an important manifestation of malnutrition, sarcopenia is a condition characterized by loss of muscle mass and strength and is associated with poor prognosis and even mortality in patients undergoing maintenance hemodialysis (MHD), with an incidence ranging from 13.7% to 37.3% [1–3]. The causes of it are multifactorial and complicated. Routine indicators currently used to assess sarcopenia include handgrip strength (HGS), skeletal muscle mass, calf circumference, muscle cross-sectional area, the chair stand test, and gait speed [4]. HGS measurement is a repeatable and trustworthy method for evaluating muscle strength as well as an indicator of general health and nutritional status and has been identified as a readily available parameter of muscle status [5–8]. HGS was reportedly associated with sarcopenia, inflammation, malnutrition and mortality in dialysis patients [2, 9, 10]. In turn, inflammation and nutritional status also affect HGS [11].

As a potentially essential element for human health, nickel plays a dual role in the human body. On one hand, nickel is crucial for the normal physiological activities [12]. On the other hand, adverse effects could be attributable to nickel exposure [12, 13]. There is currently no consensus on whether nickel level in dialysis population is deficient, normal, or elevated. Earlier studies have reported that compared with the healthy control group, hemodialysis patients have higher levels of nickel in their lymphocytes and higher levels of plasma nickel, which may be related to the possibility of early dialysis fluid contamination by nickel, causing nickel to transfer to patients [14–16]. However, a meta-analysis showed that there was no significant difference in plasma, blood, or serum nickel between the healthy control group and dialysis patients [17].

Noteworthily, previous animal researchers found that nickel could affect muscle development [18, 19]. In addition, a recent study reported that plasma nickel level correlated with low muscular strength measured with the handgrip test in non-dialysis patients, thus suggesting a possible link between these two parameters [20]. Nevertheless, to the best of our knowledge, association between blood nickel and HGS in patients undergoing MHD has not been elucidated yet. Therefore, we carried out this cross-sectional study to investigate the hypothesis that blood nickel was related to HGS in patients undergoing MHD.

## Materials and methods

### Study population

In this single-center cross-sectional study, patients who regularly underwent hemodialysis treatment at the hemodialysis center of Jiangmen Central Hospital in October 2021 were enrolled. Eligibility criteria were as follows: (1) hemodialysis therapy for more than 3 months, 3 times a week, 4 h per visit; (2) aged ≥ 18 years; (3) a signed informed consent form; and (4) with self-awareness, ability to complete HGS measurement. The exclusion criteria were as follows: (1) a history of severe infection, or trauma within 3 months; (2) definite diagnosis of malignancies; and (3) refusal to participate in the study. The research program conformed to the Helsinki Declaration and was approved by the Ethics Committee of the Jiangmen Central Hospital (approval number 202167).

### Demographic, clinical, and laboratory parameters

The following demographic and clinical parameters were collated: age, sex, height, dry weight, dialysis vintage, Charlson comorbidity index [21], hypertension, primary kidney disease, and body mass index (calculated as BMI = dry weight/height^2^). Venous blood samples were collected prior to the hemodialysis session and were analyzed in the clinical laboratory department of our hospital to determine the following parameters: white blood cell count, hemoglobin, serum C-reactive protein, blood urea nitrogen, serum creatinine, serum albumin, serum triglyceride, serum total cholesterol, serum high density lipoprotein, serum low density lipoprotein, serum calcium, serum phosphorus and serum iPTH (intact parathyroid hormone). The Kt/V (urea clearance index) was used to quantify the adequacy of dialysis treatment, where K is dialyzer clearance rate of urea; t is the dialysis time; and V is the volume of distribution of urea. The above laboratory parameters were routine tests conducted in our center every three months.

### Determination of nickel

The blood level of nickel was detected using inductively coupled plasma mass spectrometry (ICP-MS, Agilent 7900, Agilent Technologies Inc, California, US).

### Anthropometrical parameters

HGS was measured by a Harpenden handgrip dynamometer (Weighing Apparatus Group Co., Ltd, Xiangshan, Guangdong, China) in the dominant hand or the hand without a fistula if implanted [1, 22]. Every participant squeezed the device with the maximal isometric force and maintained this force for 5 s. Measurements of HGS were repeated thrice, and a maximum value was recorded. We measured HGS to determine muscle strength. Skinfold thickness, upper arm circumference, hip circumference, and waist circumference were measured according to Frisancho [23]. Using a skinfold caliper (Lange Skinfold Caliper Beta Technology Inc., Cambridge, MD) to measure triceps skinfold thickness, and the average value was recorded. Upper arm circumference, hip circumference, and waist circumference were measured with a plastic tape measure (Plum Flower, Zhejiang, China).

### Statistical analyses

Descriptive statistics comprised the mean ± standard deviation for continuous variables with normal distribution, median (25%, 75% interquartile range) for data with a skewed distribution, and percentages (%) for categorical variables. Eligible patients were divided into three groups by the tertile of the blood nickel level. Tertile 1 represented for blood nickel level ≥ 5.2 ug/L; tertile 2 represented for blood nickel level < 5.2 ug/L and ≥ 4.5 ug/L; and tertile 3 represented for blood nickel level < 4.5 ug/L. Differences among the three groups were generated using one-way analysis of variance, the Kruskal–Wallis test, or the χ^2^ test, as appropriate. Spearman’s analyses were used to evaluate correlations between blood nickel level and HGS level, as well as some clinical parameters. Univariate and multivariate linear regressions analyses were performed to analyze association of blood nickel level and HGS in patients undergoing hemodialysis. We also employed the restricted cubic spline regression model to evaluate nonlinearity. All analyses were performed using SPSS version 22.0 (IBM Corp., Armonk, NY, USA) and the R package 3.6.0 (https://www.r-project.org/). Statistical significance was set at *p* value < 0.05.

## Results

**Fig. 1:**
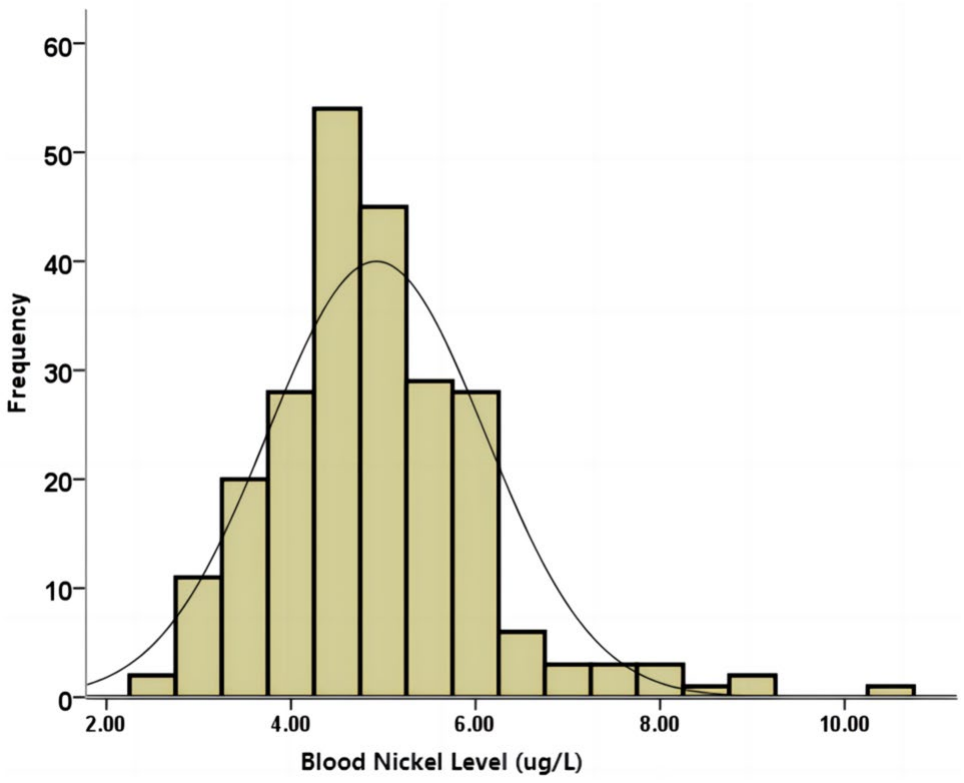
Distribution of blood nickel level

A total of 236 (men: women = 151:85) MHD patients, with an average age of 55.51 ± 14.27 years, were enrolled in the study. The median dialysis vintage was 83 (IQR: 48 to 125) months. The primary cause of CKD was glomerulonephritis (*n* = 143, 60.6%), followed by diabetic nephropathy (*n* = 40, 16.9%) and hypertensive nephropathy (*n* = 18, 7.6%). In these patients, the blood nickel level ranges from 2.5 to 10.5 ug/L, all of which were within normal limits. The mean level of the blood nickel level was 4.92 ± 1.78 ug/L, and showed a normal distribution (Fig. 1).

### Comparisons of clinical parameters between groups based on the blood nickel level

**Fig. 2:**
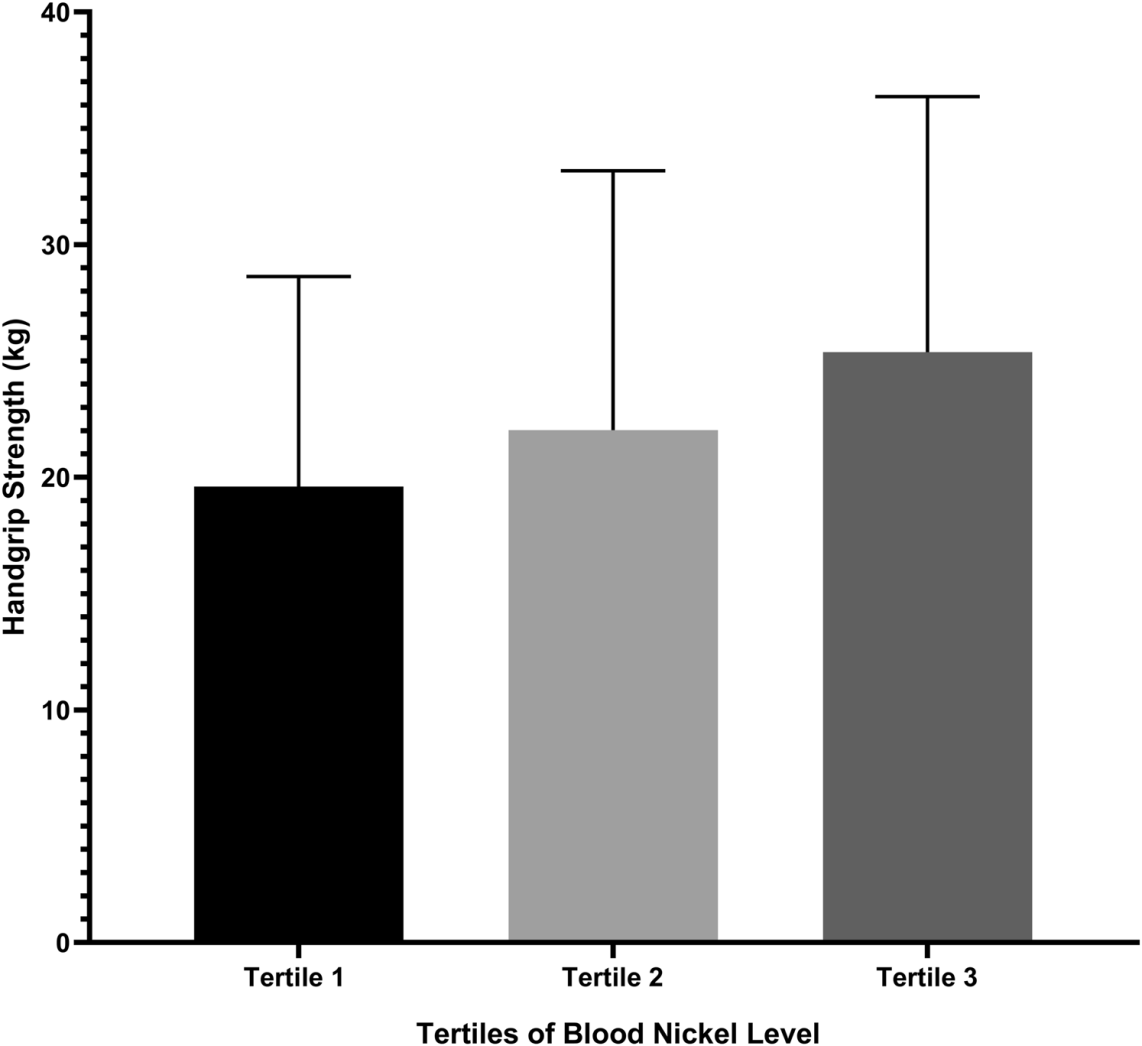
Handgrip strength level between groups based on the tertiles of blood nickel level

According to the tertiles of blood nickel level, the study participants were divided into three groups: tertile 1 group (blood nickel level ≥ 5.2 ug/L); tertile 2 group (4.5 ug/L ≤ blood nickel level < 5.2 ug/L); and tertile 3 group (blood nickel level < 4.5 ug/L). Patients in group with a higher blood nickel level tended to be female, had longer dialysis vintage and higher Kt/V, but lower BMI, serum creatinine, hemoglobin, and HGS level (all *p* value < 0.05; Table 1). Compared to the tertile 1 group, the tertile 2 and tertile 3 group had a significantly higher HGS level (19.5 ± 9.0 kg vs. 22.0 ± 11.2 kg vs. 25.4 ± 11.0 kg, respectively; *p* value = 0.002; Fig. 2).

**Table 1:** Characteristics of the total study population and after dividing by tertiles of blood nickel level (ug/L)

Variables	Total (*n* = 236)	Tertile 1 (blood nickel level ≥ 5.2 ug/L) (*n* = 77)	Tertile 2 (4.5 ug/L ≤ blood nickel level < 5.2 ug/L) (*n* = 77)	Tertile 3 (blood nickel level < 4.5 ug/L) (*n* = 82)	*p* value
Demographics					
Age (years)	55.5 ± 14.3	54.0 ± 14.0	57.7 ± 14.3	54.9 ± 14.5	0.245
Sex, female *n* (%)	85 (36.0)	47 (57.3)	23 (29.9)	15 (19.5)	< 0.001
Dialysis vintage(months)	90.5 ± 53.0	104.2 ± 51.2	94.1 ± 53.6	72.0 ± 49.6	< 0.001
Primary nephropathy					
Glomerulonephritis, *n* (%)	143 (60.6)	40 (51.9)	48 (62.3)	55 (67.1)	0.374
Diabetic nephropathy, *n* (%)	40 (16.9)	19 (24.7)	13 (16.9)	8 (9.8)	
Hypertensive nephropathy, *n* (%)	18 ( 7.6)	7 (9.1)	3 (3.9)	8 (9.8)	
Modified CCI	2 (2, 3)	2 (1, 3)	2 (1, 3)	2 (2, 3)	0.398
Hypertension, n (%)	154 (86.5)	51 (87.9)	49 (81.7)	54 (90)	0.380
BMI (kg/m^2^)	21.1 ± 3.4	19.7 ± 3.2	21.3 ± 2.7	22.5 ± 3.5	< 0.001
Laboratory parameters before dialysis sessions					
Hemoglobin (g/L)	107.4 ± 15.2	103.6 ± 16.2	110.5 ± 14.8	108.5 ± 13.9	0.014
White blood cell (10^9^)	6.5 ± 2.1	6.3 ± 2.2	6.5 ± 1.9	6.7 ± 2.0	0.478
C-reactive protein (mg/L)	3.0 (1.3, 6.4)	2.9 (1.3, 6.6)	3.3 (1.5, 6.1)	2.3 (1.1, 6.0)	0.541
Serum albumin (g/L)	40.6 ± 4.0	40.2 ± 5.5	40.7 ± 3.0	40.9 ± 3.2	0.587
Cholesterol (mmol/L)	3.8 ± 1.0	4.0 ± 0.9	3.9 ± 1.0	3.7 ± 1.0	0.161
Triglyceride (mmol/L)	1.9 ± 1.4	1.7 ± 1.2	2.0 ± 1.5	1.8 ± 1.5	0.370
High density lipoprotein (mmol/L)	1.1 ± 0.4	1.2 ± 0.3	1.1 ± 0.3	1.1 ± 0.4	0.065
Low density lipoprotein (mmol/L)	2.2 ± 0.8	2.3 ± 0.7	2.3 ± 0.8	2.1 ± 0.8	0.232
Calcium (mmol/L)	2.3 ± 0.3	2.3 ± 0.3	2.2 ± 0.2	2.3 ± 0.2	0.399
Phosphorus (mmol/L)	2.1 ± 0.6	2.1 ± 0.6	2.1 ± 0.6	2.2 ± 0.7	0.495
iPTH (pmol/L)	269.0 (159.4, 395.7)	248.2 (141.4, 411.2)	270.7 (151.2, 365.5)	269.0 (174.8, 399.8)	0.620
Uric acid (μmol/L)	435.0 (379.5, 507.5)	428.0 (383.5, 494.0)	452.0 (380.0, 538.5)	431.0 (371.0, 501.0)	0.506
Urea nitrogen (mmol/L)	28.8 ± 9.0	26.4 ± 9.2	29.5 ± 8.9	30.2 ± 8.6	0.037
Serum creatinine (μmol/L)	1031.0 (811.2, 1188.0)	1064.0 (850.6, 1229.0)	1072.0 (810.3, 1251.0)	924.0(797.6, 1095.0)	0.020
Kt/V	1.4 ± 0.3	1.6 ± 0.3	1.3 ± 0.3	1.3 ± 0.2	< 0.001
Parameters of anthropometric measurement					
Handgrip strength (kg)	22.2 ± 10.7	19.5 ± 9.0	22.0 ± 11.2	25.4 ± 11.0	0.002
Upper arm circumference (cm)	26.1 ± 3.9	25.3 ± 2.8	26.8 ± 3.6	26.2 ± 4.9	0.151
Skinfold thickness (cm)	8.2 ± 5.0	8.1 ± 5.3	7.6 ± 4.5	8.7 ± 5.1	0.573
Hip circumference (cm)	90.1 ± 6.9	88.4 ± 6.9	91.2 ± 7.5	91.1 ± 6.0	0.079
Waist circumference(cm)	82.8 ± 11.3	80.0 ± 11.6	83.9 ± 10.0	84.6 ± 11.8	0.098

*CCI* Charlson comorbidity index, *BMI* body mass index, *iPTH* intact serum parathyroid hormone, *Kt/V* K dialyzer clearance of urea, *t* dialysis time, *V* volume of distribution of urea

### Correlation analyses between blood nickel level, handgrip strength level and clinical parameters

**Fig. 3:**
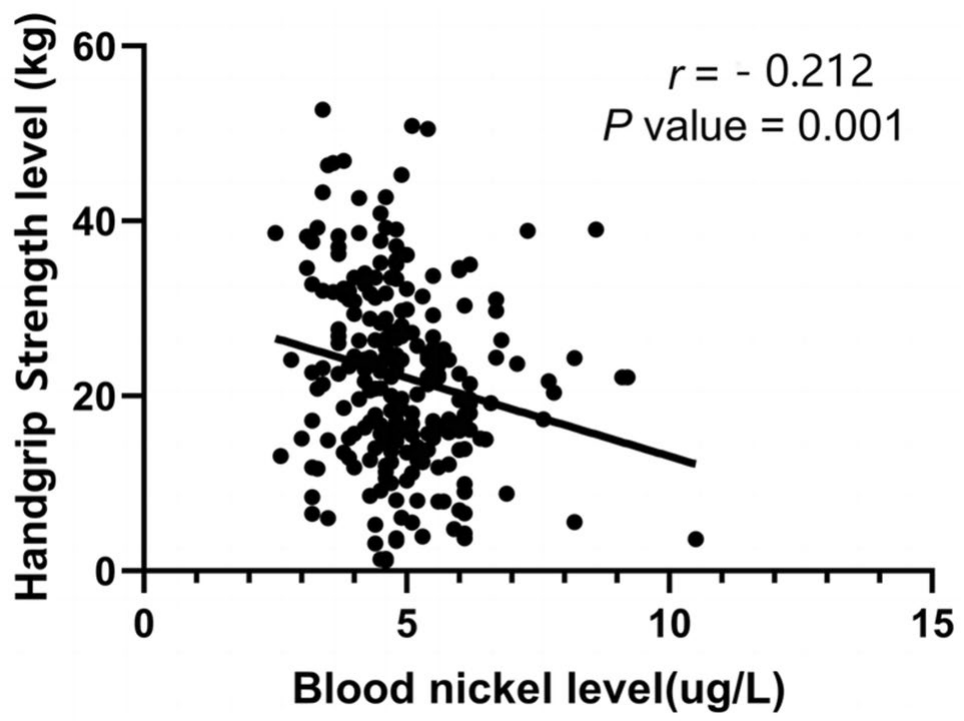
Correlation between handgrip strength level and blood nickel level

In the Spearman analyses, blood nickel level was positively correlated with duration of dialysis, whereas it was negatively correlated with BMI, HGS, upper arm muscle circumference, hip circumference, waist circumference, hemoglobin, serum creatinine (all *p* value < 0.05; Table 2, Fig. 3). We also found that HGS was positively correlated with BMI, upper arm muscle circumference, hemoglobin, serum albumin, serum creatinine, triglyceride, phosphorus, but negatively correlated with age and C-reactive protein. (All *p* value < 0.05; Table 2).

**Table 2:** Correlations between blood nickel level, handgrip strength level and clinical parameters

	Blood nickel level (ug/L)		Handgrip strength level (kg)	
	*r*	*p* value	*r*	*p* value
Age (years)	− 0.043	0.514	− 0.509	< 0.001
Dialysis vintage (months)	0.296	< 0.001	− 0.099	0.128
BMI (kg/m^2^)	− 0.394	< 0.001	0.176	0.014
Handgrip strength (kg)	− 0.212	0.001	NA	NA
Upper arm circumference (cm)	− 0.209	0.012	0.198	0.018
skinfold thickness (cm)	− 0.068	0.423	0.026	0.760
Hip circumference (cm)	− 0.230	0.006	0.095	0.265
Waist circumference(cm)	− 0.214	0.011	− 0.075	0.381
Hemoglobin (g/L)	− 0.131	0.045	0.195	0.003
Serum albumin (g/L)	− 0.036	0.591	0.284	< 0.001
C-reactive protein (mg/L)	− 0.031	0.648	− 0.207	0.002
Serum creatinine (μmol/L)	− 0.149	0.022	0.528	< 0.001
Uric acid (μmol/L)	− 0.036	0.593	0.064	0.342
Cholesterol (mmol/L)	0.107	0.112	− 0.009	0.895
Triglyceride (mmol/L)	− 0.013	0.843	0.179	0.007
Calcium (mmol/L)	0.038	0.564	0.026	0.694
Phosphorus (mmol/L)	− 0.064	0.333	0.207	0.001
iPTH (pmol/L)	− 0.060	0.367	− 0.054	0.421

*BMI* body mass index, *iPTH* intact serum parathyroid hormone

### Association between blood nickel level and handgrip strength

**Fig. 4:**
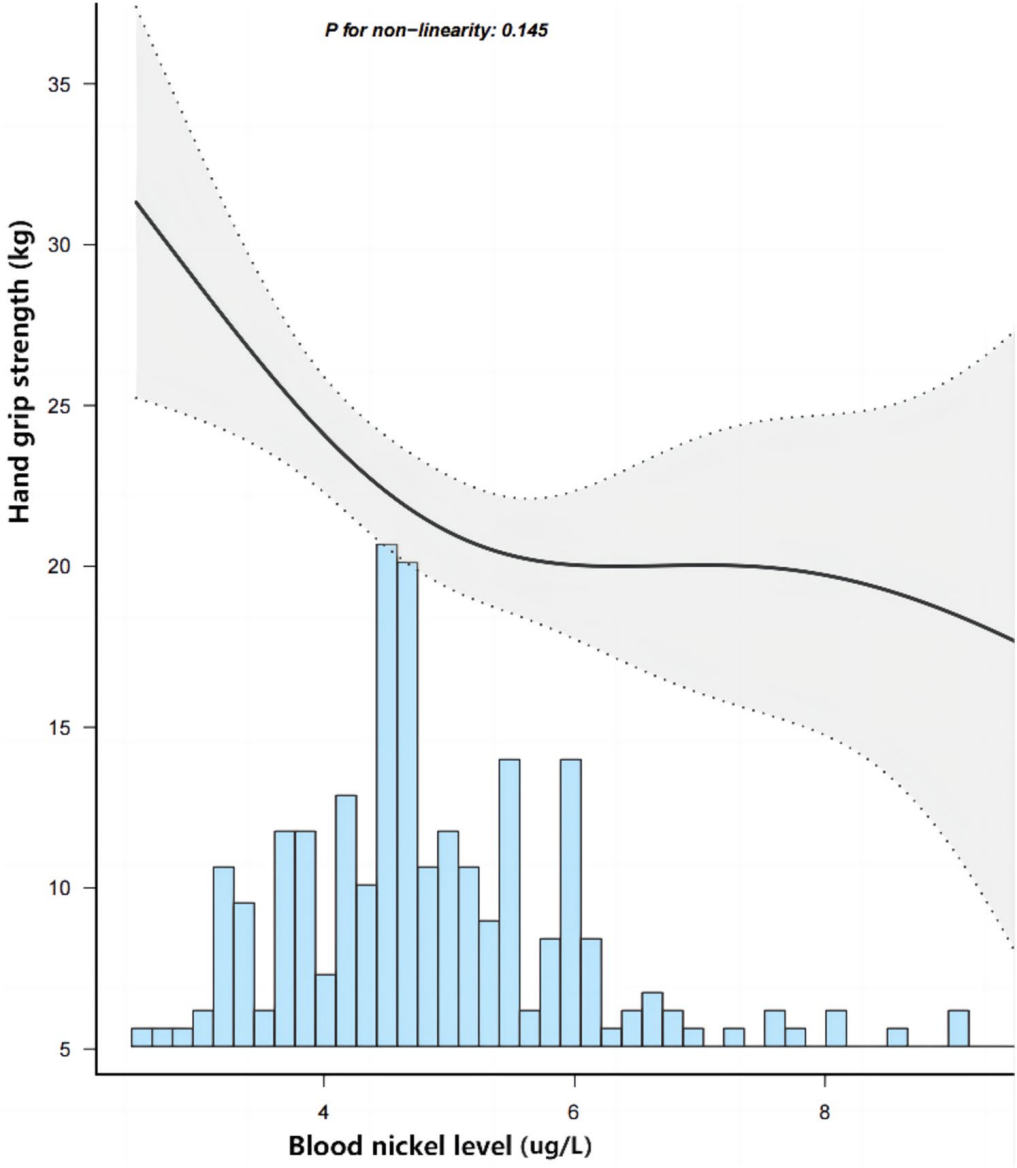
Handgrip strength level in MHD patients along with the change of the blood nickel level from the restricted cubic spline model

Table 3 displays the results obtained from multivariate linear regression analyses. The results demonstrated that the blood nickel level was independently associated with the HGS level in different models. After a full adjustment for age, sex, dialysis vintage, modified CCI, hypertension, total Kt/V, BMI, upper arm circumference, hip circumference, waist circumference, serum creatinine, uric acid, white blood cell, hemoglobin, albumin, triglyceride, cholesterol, serum calcium, phosphorus, iPTH, and C-reactive protein, we found that for every 1ug/L increase in nickel level, the patient’s HGS decreases by 2.81 kg (*β*: − 2.810, 95% confidence interval: − 5.036 to − 0.584, *p* = 0.014). In addition, restricted cubic spline was performed, and the result showed that the relationship between the blood nickel level and HGS level was nearly linear. (Fig. 4).

**Table 3:** Result of multiple linear regression models relating the blood nickel level (independent variable) with the handgrip strength level (dependent variable)

	Model	Handgrip strength level (kg)		
		*β*	95% confidence interval	*p* value
Blood nickel level (ug/L)	Model 1	− 1.798	− 2.941, − 0.656	0.002
	Model 2	− 2.021	− 3.441, − 0.602	0.006
	Model 3	− 1.949	− 3.706, − 0.192	0.030
	Model 4	− 2.810	− 5.036, − 0.584	0.014

Model 1: unadjusted

Model 2: age, sex, dialysis vintage, modified CCI, hypertension, total Kt/V

Model 3: age, sex, dialysis vintage, modified CCI, hypertension, total Kt/V, BMI, serum creatinine, uric acid, white blood cell, hemoglobin, albumin, triglyceride, cholesterol, serum calcium, phosphorus, iPTH, and C-reactive protein

Model 4: age, sex, dialysis vintage, modified CCI, hypertension, total Kt/V, BMI, serum creatinine, uric acid, white blood cell, hemoglobin, albumin, triglyceride, cholesterol, serum calcium, phosphorus, iPTH, C-reactive protein and upper arm circumference, hip circumference, waist circumference

*CCI* Charlson comorbidity index, *Kt/V* K dialyzer clearance of urea, *t* dialysis time, *V* volume of distribution of urea, *BMI* body mass index, *iPTH* intact serum parathyroid hormone

## Discussion

Sarcopenia is a widespread health problem among people on dialysis. Insufficient food intake due to loss of appetite and diet restrictions, and an increased catabolic state, including persistent inflammation, metabolic acidosis, hormonal imbalance, and physical inactivity, leads to excessive muscle wasting, attributing to a considerable risk of adverse clinical prognoses [24–27]. The diagnosis of sarcopenia requires the presence of low muscle mass, along with either low grip strength or low physical performance [28]. Among the assessment measure of muscle status, HGS measurement is an easily available one. Given that HGS may be an independent predictor of sarcopenia [29], and was also reportedly associated with poor prognosis in dialysis patients, it is of great significance to explore the relative factor of HGS.

Nickel can enter the human body through the respiratory tract, digestive tract, and skin, and can be excreted from the body through feces and urine. The main sources of nickel in food are vegetables and seafood. In our study, the blood nickel level ranges from 2.5 to 10.5 ug/L, all of which were within normal limits. The results of our study support our hypothesis that blood nickel level was negatively correlated with HGS in patients undergoing hemodialysis. After adjusted for some confounding factors, we also found that for every 1ug/L increase in blood nickel level, the patient’s HGS decreases by 2.81 kg. Our results were in accordance with previous study [20], which showed significant associations between plasma nickel level and HGS in other population.

Recently, animal studies also found that nickel might affect muscle metabolism. After nickel pellet implantation, the gene expression in mouse muscle changed over time, which had a negative impact on energy metabolism [18]. Another study indicated that nickel might affect muscle development by disrupting calcium-dependent myogenesis in developing oriental fire-bellied toad embryos [19]. Besides, there was also research reported that nickel might induce hyperglycemia and glycogenolysis and affect the antioxidant system in white muscle of goldfish [30].

The possible explanations for the correlation between higher blood nickel concentration and lower handgrip strength in dialysis patients are intriguing. One possible explanation is that bivalent nickel ions have ability to compete with calcium signaling in skeletal muscles. There have been reports suggesting that nickel can block certain calcium channels [31–33], particularly T-type voltage-gated bivalent calcium ions channels [34, 35], as also demonstrated in smooth muscle in preclinical models [35, 36]. Another potential mechanism behind this correlation involves the inhibition of muscle glutathione reductase activity by nickel, leading to an imbalance of the antioxidant system, which can result in muscle damage [30]. Therefore, it is possible that high levels of nickel in the blood may negatively affect the skeletal muscle function in dialysis patients by disrupting calcium signaling and/or causing oxidative stress.

The potential effect of nickel on calcium-sensing receptor (CaSR) and parathyroid gland function might be another explanation of the association of nickel and sarcopenia. As nonspecific calcium channel blocker, nickel might affect the function of parathyroid cells in vitro experiments [37]. We suspected that it might passivate the sensitivity of CaSR to extracellular calcium ions, simulate hypocalcemia, and promote the rapid release and secretion of endogenous parathyroid hormone(PTH). Animal study reported that administration of PTH might impair energy production and utilization and influence protein metabolism in skeletal muscle [38, 39]. Elevated PTH was also associated with lower muscle mass [40] and sarcopenia [41]. Therefore, it is possible that nickel may affect the CaSR and parathyroid gland function so that skeletal muscle function might be affected in dialysis patients.

At present, this is the first study to focus on hemodialysis patients to evaluate the relationship of blood nickel and HGS. To some extent, our study is convincing because we used multivariate linear regression analysis. However, several limitations should be acknowledged. First, we could not determine the causal relationship between blood nickel and HGS because this is a cross-sectional single-center study. Second, the study sample size was relatively small; thus, bias was inevitable. Third, blood nickel level was measured only once while the patients were enrolled in the study. Dynamically monitoring how blood nickel changes is worthy of further research. Finally, we did not record parameters related to oxidative stress and dietary status, which may affect HGS.

## Conclusion

This study demonstrated association between higher blood nickel concentrations and lower HGS in hemodialysis patients. We speculate that the main reason is that nickel may affect muscle contraction by inhibiting calcium-dependent myogenesis, lead to imbalance of the antioxidant system and cause adverse impact on muscular energy metabolism, but the mechanism underlying is intricate. Exploring the range of blood nickel level suitable for hemodialysis patients and methods to reduce blood nickel level, which may improve muscle strength in patients undergoing MHD, deserve further research.

## Data Availability

The data sets generated during and/or analyzed during the current study are available from the corresponding author on reasonable request.
